# Allergic Bronchopulmonary Mycosis Caused by Mucor Overlapping With Invasive Pulmonary Mucormycosis: A Case Report

**DOI:** 10.3389/fmed.2022.831213

**Published:** 2022-02-24

**Authors:** Ruhui Zhang, Ge Jin, Yasheng Zhan, Lisha Shen, Yake Yao, Qiqi Gao, Qing Yang, Jianying Zhou, Hua Zhou

**Affiliations:** ^1^Department of Respiratory and Critical Care Medicine, The First Affiliated Hospital, Zhejiang University School of Medicine, Hangzhou, China; ^2^Department of Pathology, The First Affiliated Hospital, Zhejiang University School of Medicine, Hangzhou, China; ^3^State Key Lab for Diagnostic and Treatment of Infectious Diseases, First Affiliated Hospital, Zhejiang University, Hangzhou, China

**Keywords:** allergic bronchopulmonary mycosis, mucor, invasive pulmonary mucormycosis, glucocorticoid, IgE

## Abstract

Mucormycosis is a rare and invasive fungal infection with high mortality. Cases of invasive pulmonary mucormycosis that involve allergic reactions such as allergic bronchopulmonary mycosis are rarely reported. Herein, we describe a case of invasive pulmonary mucormycosis overlapping with allergic diseases in a patient who presented with eosinophilia and high total plasma immunoglobulin E (IgE). The patient was successfully treated with systemic corticosteroids (initial dose of prednisolone approximately 0.5 mg/kg per day, total duration less than 3 months) combined with posaconazole antifungal therapy. The treatment resulted in recovery of peripheral-blood eosinophil count and total plasma IgE, and significant reduction in lung lesions. A subsequent lobectomy was performed. The findings in this case indicate that systemic corticosteroid therapy may contribute to the treatment of pulmonary mucormycosis combined with allergic factors.

## Introduction

A pulmonary fungal infection can simultaneously cause both invasive infection and allergic reactions such as allergic bronchopulmonary mycosis (ABPM), and this can be associated with undesirable therapeutic effects and poor outcomes (1). Pulmonary mucormycosis is an invasive fungal disease with high mortality (2). Herein, we describe a case of invasive pulmonary mucormycosis with concurrent allergic reaction in a patient who presented with eosinophilia and high plasma IgE, and in whom treatment with systemic corticosteroid (SCS) combined with posaconazole (antifungal therapy) achieved a favorable outcome.

## Case Description

A previously well 38-year-old man with protracted cough, sputum, and chest pain was diagnosed with pulmonary mucormycosis *via* a lung puncture. He had no history of diabetes mellitus, HIV infection, atopy, or any other disease. Chest computed tomography revealed a mass in the right upper lobe of the lung involving the right clavicular region, axillary mediastinum, hilar lymph node, and chest wall. A lung biopsy was performed and indicated pulmonary mucormycosis. Posaconazole oral suspension (200 mg every 6 h) was started on day 19.

**Figure 1:**
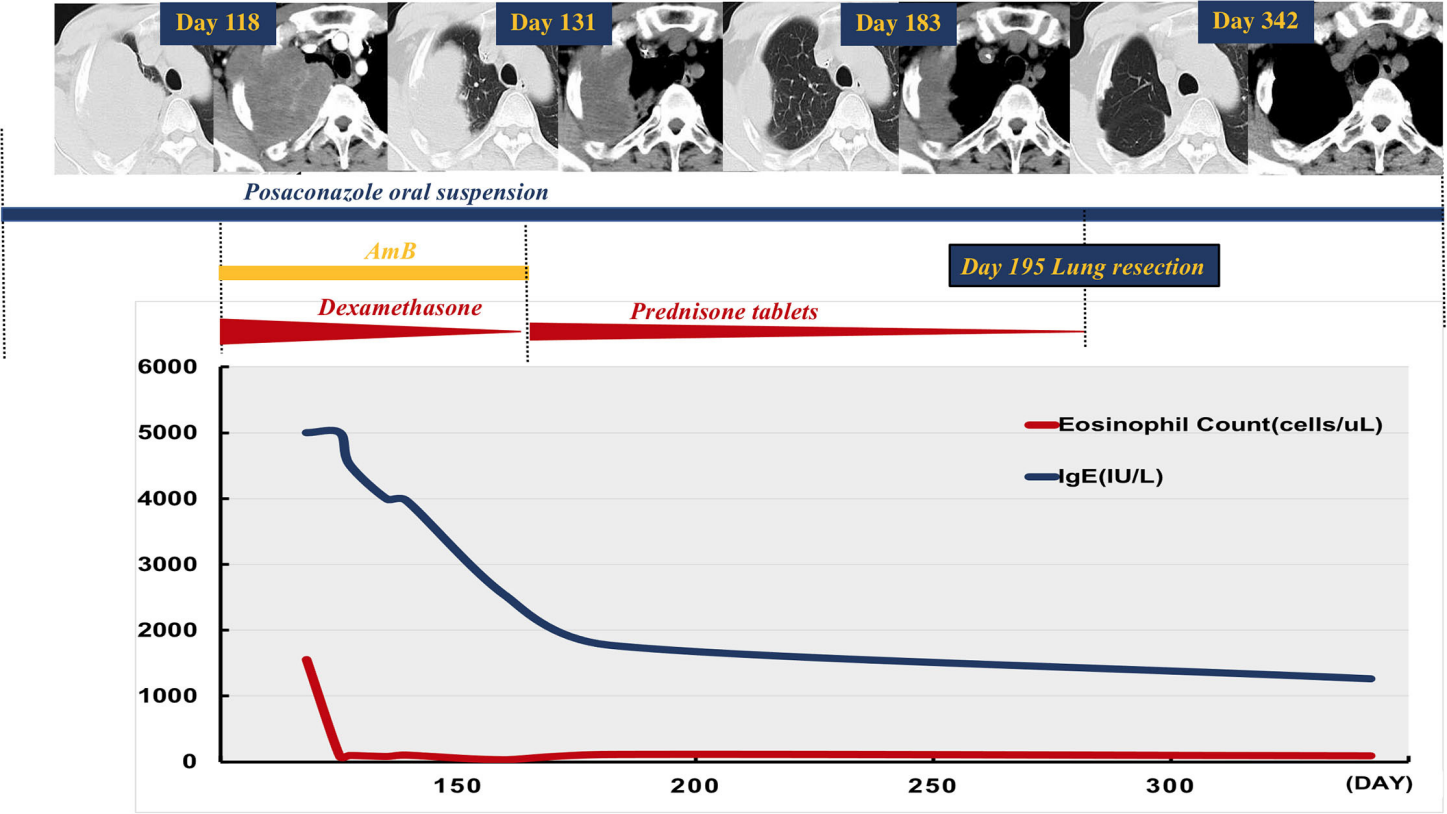
Case time line. Chest computed tomography on day 118 depicted a mass in the right upper lobe of the lung involving the right chest wall, and there was no reduction in its size after 3 months of treatment with oral posaconazole suspension. Concurrent amphotericin B and dexamethasone were started after repeat diagnostic lung biopsy on day 120. Amphotericin B was stopped on day 128. Systemic corticosteroid (SCS, prednisone tablets) was given after repeat imaging on day 131 and depicted a marked reduction in mass size. Radiology on day 183 indicated substantial improvement in the lung lesions with concurrent weaning of SCS. The patient underwent subsequent lobectomy on day 195, and posaconazole was used during the following 3 months. He was well, with no radiological evidence of relapse on day 342 follow-up. Reduction in peripheral eosinophil count and total serum IgE level was observed after initial SCS and anti-fungal therapy.

**Figure 2:**
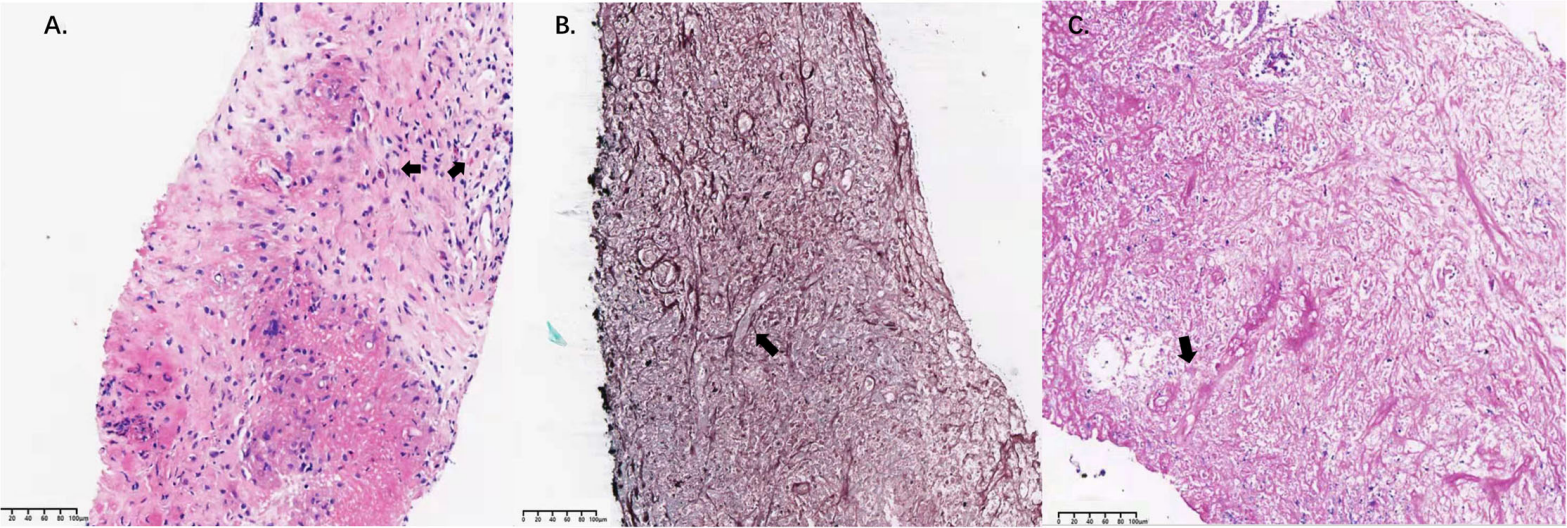
Pathological findings in lung tissue samples. **(A)** High-magnification image of a hematoxylin-eosin-stained specimen (bar = 100 μm). Infiltration of eosinophils was observed (arrow). **(B)** Gomori's methenamine silver staining revealed filamentous fungi with broad and nonseptate hyphae in the lung lesion (arrow; bar =100 μm). **(C)** Periodic acid Schiff staining revealed filamentous fungi with broad and nonseptate hyphae in the lung lesion (arrow; bar = 100 μm).

Radiological assessment on day 118 indicated no reduction in the size of the mass after 3 months of posaconazole therapy (Figure 1). Blood tests revealed a white blood cell count of 18,100/μl (neutrophils 6,730/mm^3^, lymphocytes 1,910/mm^3^, and eosinophils 1,550/mm^3^), a platelet count of 278,000/μl, and a hemoglobin value of 11.4 g/dl. C-reactive protein level was 28.9 mg/L (normal range 0–8 mg/L). Total IgE level was > 5,000 kU/L (normal range 0–100 kU/L). Bronchoscopy revealed stenosis of the apical segment of the upper lobe, with no mucus plug. Repeated lung puncture on day 118 indicated granulomatous lesions with infiltration of a large number of lymphocytes and eosinophils. Immunofluorescence staining suggested mucormycosis infection (Figure 2). Tissue culture and metagenomic next-generation sequencing both indicated *Rhizopus* infection. Amphotericin B (0.7 mg/kg per day) with concurrent intravenous dexamethasone (5 mg per day) was started on day 120 because of failure to respond to posaconazole, but amphotericin B was stopped on day 128 after a total dose of 170 mg because of renal dysfunction.

Repeat imaging on day 131 revealed a marked reduction in the size of the mass (Figure 1). Given the eosinophilia and elevated serum IgE, we hypothesized that an allergic reaction had favored disease progression. Accordingly, SCS was administered as follows: prednisone tablets 0.5 mg/kg for 1 week, followed by 0.25 mg/kg for 3 weeks, then tapered by 5 mg every week continued for a total duration of 2 months. IgE level and eosinophil count in peripheral blood decreased during treatment with SCS and posaconazole oral suspension. Follow-up radiology on day 183 indicated substantial improvement in the lung lesions (Figure 1). The patient underwent subsequent right upper lobe lobectomy on day 195, and post-surgery he was administered a further 3 months of posaconazole treatment. The patient was well with no clinical or radiological evidence of relapse on day 342 follow-up (Figure 1).

## Discussion

Concurrent invasive pulmonary mucormycosis and host allergic reaction is relatively rare. A systematic PubMed search suggested that to date only a few case reports have described pulmonary mucormycosis with ABPM or increased eosinophil count and total IgE level (3–5). This patient exhibited increased total serum IgE (> 1,000 IU/ml) and peripheral blood eosinophilia (> 500/mm^3^). Together with substantial infiltration of eosinophils in mucoraceous lesions, these observations indicated an allergic response triggered by *Mucor* that was poorly responsive to conventional antifungal therapies. We postulate that this patient probably developed ABPM during the course of pulmonary mucormycosis based on the modified International Society for Human and Animal Mycology working group criteria (6, 7). Notably however, the definition and diagnostic criteria of ABPM are still controversial (8).

The management of ABPM includes two main components, SCS anti-inflammatory therapy and antifungal therapy, to alleviate fungal burden (9). Although there are no previous reports on successful SCS treatment for mucormycosis-induced ABPM (3–5), there is emerging evidence that SCS combined with immunotherapy is effective in patients with invasive fungal infections associated with T-helper 2 immune responses. Anti-interleukin 5 (mepolizumab) therapy can effectively dampen aberrant T-helper 2 responses, and may be a new therapeutic option for invasive pulmonary mycosis (10). In this patient, low-dose and medium-dose SCS and concurrent posaconazole treatment rapidly achieved symptomatic control, reduced the size of the lung mass, and suppressed peripheral blood eosinophilia. We assume that the major response to SCS was mediated by suppression of eosinophilic inflammation in invasive pulmonary mucormycosis. In a randomized controlled trial of ABPM, it was reported that a median dose (0.5 mg/kg per day) of prednisolone treatment may be as effective as or safer than a higher dose (0.75 mg/kg per day); hence a lower dose and a shorter duration may be better options (11).

This report has several limitations, which include the absence of amphotericin B liposome therapy and posaconazole therapeutic drug monitoring due to drug availability. The patient only received treatment with posaconazole suspension, because posaconazole tablets and intravenous formulation were not marketed, and because liposomal amphotericin B was not available in China at the time. Global mucormycosis guidelines recommend liposomal amphotericin B as first-line treatment. In clinical studies, plasma concentrations in patients administered with sustained-release posaconazole tablets and intravenous formulations were higher than those in patients administered with oral suspensions (12, 13). We speculate that this patient had ABPM caused by *Mucor* based on clinical evidence. Direct evidence of allergy against Mucorales such as mucor-specific IgE was not obtained because of experimental limitations. We concluded that there was clear evidence of type 2 hypersensitivity (raised IgE and eosinophil count) that responded to prednisolone.

To our knowledge, this is the first report to describe the administration of SCS as part of successful therapy for invasive pulmonary mucormycosis. It also provides important clinical insight indicating that invasive pulmonary mucormycosis can be present with severe allergic reaction.

## Data Availability Statement

The original contributions presented in the study are included in the article/supplementary material, further inquiries can be directed to the corresponding author.

## Ethics Statement

This study was conducted in accordance with the Declaration of Helsinki, and reviewed and approved by the Research Ethics Committee of the First Affiliated Hospital of Zhejiang University (IIT20210130A). The patient provided written informed consent for the publication of clinical information including lung CT and pathological images.

## Author Contributions

All authors listed have made a substantial, direct, and intellectual contribution to the work and approved it for publication.

## Conflict of Interest

The authors declare that the research was conducted in the absence of any commercial or financial relationships that could be construed as a potential conflict of interest.

## Publisher's Note

All claims expressed in this article are solely those of the authors and do not necessarily represent those of their affiliated organizations, or those of the publisher, the editors and the reviewers. Any product that may be evaluated in this article, or claim that may be made by its manufacturer, is not guaranteed or endorsed by the publisher.

## Abbreviations

IgE: immunoglobin E
SCSs: systemic corticosteroids
ABPM: allergic bronchopulmonary mycosis
CT: computedtomography
PET-CT: positron emission-CT
WBC: white blood cell
CRP: C-reactive protein
AmB: amphotericin B
ISHAM: International Society for Human and Animal Mycology.

## References

[1] Obar JJ Carvalho A Vitte J Ranque S. Editorial: host and pathogen determinants of allergic and invasive fungal diseases. Front Immunol. (2020) 11:856. 10.3389/fimmu.2020.00856 32425953 PMC7205003

[2] Nucci M Engelhardt M Hamed K. Mucormycosis in South America: a review of 143 reported cases. Mycoses. (2019) 62:730–8. 10.1111/myc.12958 31192488 PMC6852100

[3] Sato M Gemma H Sano T Ono T Atsumi E Ito I. Pulmonary mucormycosis caused by Cunninghamella bertholletiae in a non-immunocompromised woman. Nihon Kokyuki Gakkai Zasshi. (2001) 39:758–62. 11828731

[4] Hirano T Yamada M Sato K Murakami K Tamai T Mitsuhashi Y. Invasive pulmonary mucormycosis: rare presentation with pulmonary eosinophilia. BMC Pulm Med. (2017) 17:76. 10.1186/s12890-017-0419-1 28454572 PMC5410085

[5] Deepak D Singh Rajput M Sharma B Chowdhary A. Allergic bronchopulmonary mycosis due to fungi other than aspergillus. Eur Ann Allergy Clin Immunol. (2019) 51:75–9. 10.23822/EurAnnACI.1764-1489.87 30832470

[6] Agarwal R Chakrabarti A Shah A Gupta D Meis JF Guleria R. Allergic bronchopulmonary aspergillosis : review of literature and proposal of new diagnostic and classification criteria. Clin Exp Allergy. (2013) 43:850–73. 10.1111/cea.12141 23889240

[7] Asano K Hebisawa A Ishiguro T Takayanagi N Nakamura Y Suzuki J. New clinical diagnostic criteria for allergic bronchopulmonary aspergillosis/mycosis and its validation. J Allergy Clin Immunol. (2020) 147: 1261–8. 10.1016/j.jaci.2020.08.029 32920094

[8] Muthu V Agarwal R. Is the “probable” category required in the diagnosis of ABPA? J Allergy Clin Immunol. (2021) 147:1119–21. 10.1016/j.jaci.2020.11.029 33419605

[9] Chowdhary A Agarwal K Kathuria S Gaur SN Randhawa HS Meis JF. Allergic bronchopulmonary mycosis due to fungi other than Aspergillus: a global overview. Crit Rev Microbiol. (2014) 40:30–48. 10.3109/1040841X.2012.754401 23383677

[10] Fracp DKY Mbbs TS Mbbs CB Burgner D Bryant PA Franzcr TMC. Refractory thoracic conidiobolomycosis treated with mepolizumab immunotherapy. J Allergy Clin Immunol Pract. (2021) 9:2527–30. 10.1016/j.jaip.2021.01.044 33601049

[11] Agarwal R Aggarwal AN Dhooria S Sehgal IS Garg M Saikia B. A randomised trial of glucocorticoids in acute-stage allergic bronchopulmonary aspergillosis complicating asthma. Eur Respir J. (2016) 47:490–8. 10.1183/13993003.01475-2015 26585431

[12] Lenczuk D Zinke-Cerwenka W Greinix H Wölfler A Prattes J Ines ZS. Antifungal prophylaxis with posaconazole delayed-release tablet and oral suspension in a real-life setting: plasma levels, efficacy, and tolerability. Antimicrob Agents Chemother. (2018) 62:1–9. 10.1128/AAC.02655-17 29581116 PMC5971564

[13] Dekkers BGJ Bakker M van der Elst KCM Sturkenboom MGG Veringa A Span LFR. Therapeutic drug monitoring of posaconazole: an update. Curr Fungal Infect Rep. (2016) 10:51–61. 10.1007/s12281-016-0255-4 27358662 PMC4896980

